# B-cell activity in children with malaria

**DOI:** 10.1186/1475-2875-11-66

**Published:** 2012-03-09

**Authors:** Jackson C Korir, Japhet K Magambo, Joseph K Mwatha, John N Waitumbi

**Affiliations:** 1 Walter Reed Project/Kenya Medical Research Institute, Kisumu, Kenya; 2 Jomo Kenyatta University of Agriculture and Technology, Nairobi, Kenya; 3 Masinde Muliro University of Science and Technology, Kakamega, Kenya; 4 Kenya Medical Research Institute, Nairobi, Kenya

## Abstract

**Background:**

Recent studies implicate deficiency of red blood cell (RBC) complement regulatory proteins (CR1 and CD55) in the pathogenesis of malarial anaemia. This study explored the involvement of B cell CD21, which has an analogous role to RBC CR1.

**Methods:**

In a case control study conducted in Kisumu District hospital, western Kenya, children with severe malaria anaemia (SMA) and those with uncomplicated malaria (UM) were assessed by flow cytometry for B cells (CD20+) numbers, expression levels of CD21 and deposition of C3dg and by ELISA for soluble CD21 (sCD21). Paired t tests were used to determine statistical significance at a = 0.05.

**Results:**

Children with SMA had significantly higher lymphocyte count (9,627.7 ± 8786.1 SD vs. 5,507 ± 2436 SD, *P *= 0.04 in the UM group) and the computed geometric mean of mature B-cell numbers based on the absolute lymphocyte count was significantly higher for SMA group: 1,823 (1,126 to 2,982, 95% CI) and 826.6 (564 to 1,220, 95% CI)] for UM group (*P *= 0.003). SMA group also had a higher percentage of CD20+ B cells (26.8 ± 9.7SD vs 20.9 ± 9.01 SD in the UM) (*P = *0.03), indicating considerable polyclonal B-cell activation. The CD21 median flourescence intensity was lower in the SMA (246.4 ± 87.4 SD vs 369 ± 137.7 SD) (*P <*0.0001), probably due to complement mediated shaving of CD21 by fixed tissue macrophages. The CD20+ B cells of SMAs had higher levels of the complement split product C3dg (18.35 ± 10 SD vs 11.5 ± 6.8 S.D), (*P *= 0.0002), confirming possible role of complement in CD21 removal. Unexpectedly, the SMAs had lower levels of sCD21 (226.5 ± 131.5 SD vs 341.4 ± 137.3 SD in the UM) (*P *< 0.0001), indicating that the shaved CD21 is not released to peripheral circulation.

**Conclusions:**

These results implicate B-cell in pathophysiology of severe malaria that involves increased B-cell proliferation, increased complement deposition and subsequent loss of membrane-bound CD21. The loss of CD21 is not by the classical enzmatic cleavage.

## Background

The global malaria burden remains enormous. According to the World Health Organization (WHO) report of 2008 [1], there were an estimated 247 million malaria cases and about 3.3 billion people at risk. Malaria has several manifestations ranging from asymptomatic parasitaemia in semi-immune individuals, to severe disease in susceptible populations that include children below five years, pregnant women and non-immune adults. In these individuals, severe malaria manifests in a variety of forms, including cerebral malaria, severe anaemia, metabolic acidosis, placental malaria and hypoglycaemia, among others [2,3]. Although these disease patterns have multiple causes, recent studies implicate acquired deficiency of RBC complement regulatory proteins such as CR1 (CD35) and CD55 in pathogenesis of severe malaria anaemia (SMA) [4,5]. These proteins protect the RBC from destruction by inhibiting formation of the C3 convertases, thereby preventing the terminal polymerization of the membrane attack complex (MAC) [6].

Similar to RBCs, B-cells also bear complement regulatory proteins such as CR1 and complement receptor 2 (CR2, CD21). These receptors have been shown to be important in the binding of opsonized IC [7-9]. Studies with C3b opsonized tetanus toxoid and opsonized immune complexes have indicated that interaction of C3 fragments with CD21 allows non-specific B cells to participate in antigen presentation [10]. Co-ligation of CD21 with B-cell receptor (BCR) by C3d-taggged antigen also promotes the trafficking of antigen to processing compartments in the B cell and for more efficient production of antigenic-peptide/class II complexes [11]. CD21 is only expressed on mature B-lymphocytes [12] and because even modest changes in the level of expression is likely to affect the capacity of B cells to respond to antigenic stimuli [13,14], the level of expression of CD21 is tightly controlled. Disease states that alter the expression of CD21 would, therefore, be expected to impact the host immunological responses.

Abnormalities of CD21 have been described in various diseases. In systemic lupus erythematosus (SLE), the expression level of both CR1 on RBCs [15] and CD21 on B cells is reduced [16]. This decline is usually disease-mediated and not genetic [17,18]. Reduced expression of CD21 has also been observed in HIV-sero-positive individuals. The effect malaria has on the expression of CD21 and the probable implication has not been explored.

CD21 also exist in a soluble form (sCD21) that is generated by shedding of the membrane-associated form and can be demonstrated in lymphocyte cultures and in plasma [19-21]. Elevated levels of sCD21 are seen in B-cell diseases such as chronic lymphocytic leukaemia (B-CLL) [22], common variable immunodeficiency (CVID), Brutons's X-lined gamma-globulinaemia [20,23] and Epstein-Bar-virus (EBV) malignancies [19].

Although proliferation of B cells is regulated, strong polyclonal B-cell response resulting in hyper-γ-globulinaemia and auto-antibodies secretion has been described in *Plasmodium falciparum *infections [24]. Studies by Donati *et al. *[25] identified the cystein-rich interdomain region 1 α (CIDR-1 α) of the *P. falciparum *erythrocyte membrane protein-1 (PfEMP-1) as responsible for the polyclonal activation. In this study, the involvement of B cells during malaria was explored by comparing the B-cell numbers, levels of complement deposition, levels of membrane bound and sCD21 in groups of children with either severe or uncomplicated malaria.

## Methods

This study was a hospital-based prospective case-control and details of the study, including ethical approvals, have been described previously [26].

### Determination of CD20+ cell population, expression level of CD21 and C3dg deposition

A 50 μL aliquot of each EDTA blood sample was placed into three wells (test sample, unstained control and isotype control) of a round-bottom, 96-well plate. To each well was added 200 μL of wash buffer (PBS containing 1% BSA and 0.1% sodium Azide). The re-suspended cells were centrifuged for three minutes at a 1,620*xg *and the supernatant discarded. Thereafter, the cell pellet was re-suspended in 100 μL of 10% mouse IgG in PBS and then incubated for 30 minutes at room temperature. This was followed by three washes and the cell pellet re-suspended in 100 μL of antibody cocktail comprising CD20PerCP (BD Pharmigen, USA)/CD21PE (BD Pharmigen, USA)/C3dgAL488 (a gift from Prof. Ronald P Taylor, University of Virginia, Charlottesville, USA) at a final concentration of 1 μg/mL and mouse IgG_1_-k for isotype control well. For the unstained control, wash buffer was added followed by incubation for 30 minutes at 4°C. Thereafter, the cells were washed and the cell pellet re-suspended in 200 μL of lysing buffer (150 mM NH_4_Cl, 10 mM NaHCO_3 _and 1 mM EDTA) for three minutes at room temperature, then washed twice. Finally, the cells were re-suspended in 100 μL of 1% Paraformaldehyde and stored at 4°C until acquisition (within 24 hours after staining).

Following staining, data acquisition was done on a Becton Dickson FACScan (BD, San Jose, CA, USA) with WinFCM (Phoenix Flow systems, San Francisco USA) as the acquisition software. Lymphocytes were gated based on FSC and SSC and 20,000 events were acquired. Analysis was done using EXPO software (Phoenix Flow systems, San Francisco USA). Expression level for CD21 and C3dg deposition were reported as median fluorescence.

### Quantification of sCD21 by ELISA

Commercially available enzyme immunoassay kits for sCD21 (Cellsciences, Massachusetts, USA) was used. Plates were supplied pre-coated with a monoclonal antibody specific for CD21. 100 μL of serially diluted standards with concentrations that ranged from 3.12-100 units/mL and 100 μL of plasma samples diluted 1:4 were added to their respective wells followed by the addition of 50 μL of a biotinylated monoclonal antibody specific for CD21 to each well and incubated for one hour at 25°C. At the end of the incubation, the wells were washed twice by adding 300 μL of the wash buffer to each well and aspirating. Thereafter, 100 μL of streptavidin-HRP was added to each well followed by 30 minutes incubation at room temperature. Unbound enzyme was removed and wells washed by adding and aspirating 300 μL of the wash buffer. 100 μL of ready-to-use 3,3', 5,5"-tetramethylbenzidine (TMB) substrate solution was added to each well and incubated at room temperature for 20 minutes in the dark. The reaction was stopped by the addition of 100 μL of 0.16 M sulfuric acid and colour intensity read at 415 nm using the VMax^® ^Kinetic ELISA Microplate Reader (Molecular devices, CA, USA). The concentration of the sCD1 in the samples was determined by extrapolating from the standard curve.

Statistical analysis was done using Graphad^® ^Prism software Version 5 (CA, USA). 2 tailed paired t-tests were performed with the level of significance set at 0.05.

## Results

### Demographics and clinical data

The demographics and clinical data for the study participants is shown in Table 1. There was no difference in the mean ages for the SMA and UM malaria group. Although the SMA group had a higher parasite density [Geometric mean (95% CI)], 33295 (19191-57764) than the UM group 22196 (10387-47432), the difference was not significant. The absolute lymphocyte count was significantly higher for the SMA group 9628 ± 8786 as compared to the UM group 5507 ± 2436 (*P *= 0.039).

**Table 1:** Demographics and clinical data of the study participants

Characteristic	Severe Anaemia	Uncomplicated Malaria	P value
Sample size (N)	30	30	

Age (months) (Mean ± SD)	16.67 ± 9.217	17.02 ± 9.5	NS

Parasitaemia [Geo mean (95% CI)]	33295 (19191-57764)	22196 (10387-47432)	NS

Hb level (g/dl, mean ± SD)	4.5 ± 1.0	8.9 ± 1.3	ND

Absolute lymphocyte count/L (mean ± S.D)	9628 ± 8786	5507.82 ± 2436	0.04

*NS*- Not significant, *ND*- Not done since Hb level was one of the recruitment criteria for SMA cases

### Mature B-cell population, expression level of CD21 and C3dg deposition in severe and uncomplicated malaria

Lymphocytes were identified on the basis of their forward scatter (FSC) and side scatter (SSC) characteristics. As shown in Table 1, absolute lymphocyte count was significantly higher in the SMA group (9628 ± 8786 SD) compared to the UM group (5,507 ± 2436 SD, *P *= 0.04).

**Figure 1:**
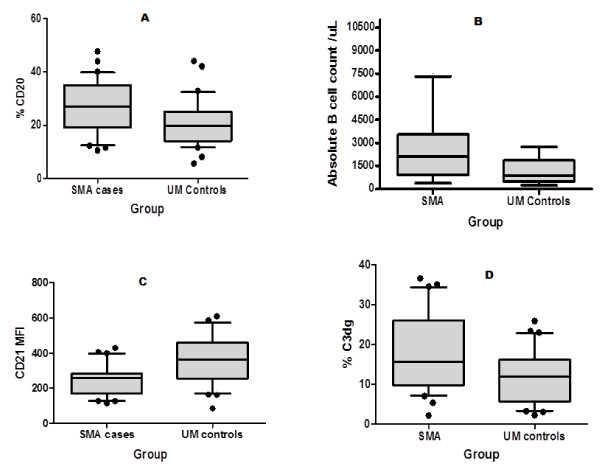
**% CD20, absolute cell count, CD21 expression level and C3dg deposition in children with severe (SMA) and uncomplicated malaria (UM)**. Panel A: Children with SMA had a higher % of CD20+ B cells compared to UM group (*P *= 0.03). Panel B: Computed absolute B-cell values were higher in children with SMA compared to those with UM (*P *= 0.003). Panel C: The median fluorescent intensity of CD21 was lower in children with SMA compared to the UM controls (*P *< 0.0001). Panel D: B cells of SMA children had a higher percentage of C3dg deposited on their surfaces compared to those in the UM group (*P *= 0.0002).

Figure 1, panel A shows the percentage CD20+ B cells in the two study groups. SMA had a higher mean percentage CD20+ cells (26.8 ± 9.7 SD) compared to the UM group (20.9 ± 9.1 SD, *P *= 0.03) and as shown in Figure 1, panel B, the computed geometric mean numbers of these mature B-cells based on the absolute lymphocyte count was 1,823 (1,126 to 2,982, 95% CI) for SMA and 826.6 (564 to 1,220, 95% CI)] for UM group (*P *= 0.003).

Figure 1, panel C shows that the SMA group had a statistically significant lower CD21 median fluorescence intensity (246.4 ± 87.4 SD) compared to the UM controls (369 ± 137.7 SD) (*P *< 0.0001). Thus although the SMA group had elevated numbers of mature B cells, their expression of membrane-bound CD21 was reduced. Lastly, as shown in Figure 1, panel D, SMAs had a higher percentage of C3dg bearing CD20+ B cells (18.35 ± 10 S.D) compared to UM control (11.5 ± 6.8 SD, *P *= 0.0002).

### Levels of plasma sCD21 in severe and uncomplicated malaria

**Figure 2:**
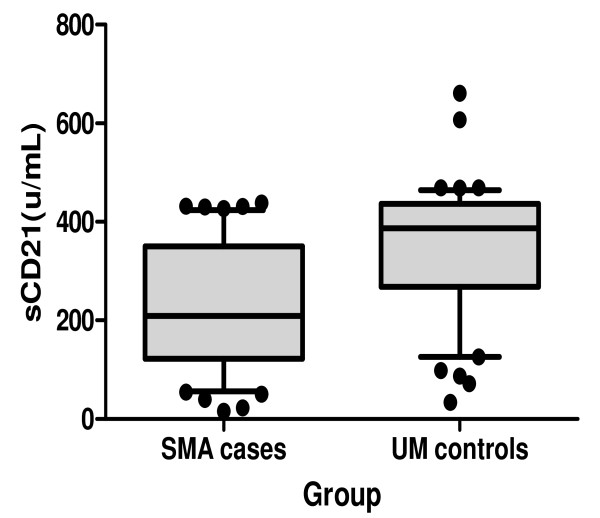
**Soluble CD21 levels during malaria**. Soluble CD21 (sCD21) were lower in the SMA group compared to UM group (*P *< 0.0001)

Plasma sCD21 was lower in SMA (246.4 ± 87.4 SD units/mL) compared to the UM controls (341.4 ± 137.3 SD, *P *< 0.0001) (Figure 2).

## Discussion

Previous studies on the pathogenesis of malarial anaemia have focused on the role of RBC complement regulatory proteins on the pathophysiology of severe malaria and have shown a marked reduction of CR1, increased deposition of complement products and increased consumption of complement in children with malarial anaemia [5,27]. Like RBC, B cell is involved in complement processing and has molecules such as CD21, also known as complement receptor 2 (CR2) that share many structural and functional characteristics with RBC CR1. The current study evaluated the effect malaria has on complement regulatory role of B cell.

As has been shown in previous studies [2,3], parasite density cannot be the key cause to severe anaemia, as the parasitaemia for children in the UM group did not differ from those with severe anaemia (Table 1). Children with SMA had increased levels of CD20+ B cells as compared to the UM (Figure 1A), with the B-computed absolute B-cell being also higher in the SMA group (Figure 1B). This expansion in B-cell population is in agreement with previous findings that have shown that B-cell population is increased during malaria [28] leading to strong polyclonal B-cell response and subsequent hyperglobulinaemia [29]. The cystein-rich inter-domain region 1α (CIDR1α) of the PfEMP1 has been incriminated as being the ligand that interacts with B cells to cause proliferation and activation [25].

Among the many roles of CD21 is to bind the complement split fragment C3dg [30], thus, like RBC CR1, mops up circulating complement products for removal by the reticuloendothelial macrophages. The removal of C3dg by macrophages inadvertently leads to shaving off of CD21 molecules, in a manner analogous to removal of RBC CR1 [31]. As shown in Figure 1C, children with SMA had lower CD21 MFI compared to those with UM. Although the mature B cells were more in SMA than in the UM, the mean level of expression of CD21 was lower (Figure 1C). Reduced CD21 expression has been reported in other diseases such as in SLE, an autoimmune disorder associated with increased levels of circulating immune-complexes [16] and in HIV sero-positive individuals [32]. Reduced expression of CD21 may have implications in the mounting of a sufficient immune response since CD21 has been shown to promote the expression of B-cell receptor (BCR) signalling through the formation of CD19/CD21 complex [33], and thus children with SMA may have impaired humoral response.

Contrary to expectation and as shown in Figure 2, the mean sCD21 was significantly lower in children with SMA compared to UM controls. The expectation would have been that the loss of membrane bound CD21 is by enzymatic cleavage and thus would end up in plasma as sCD21 [34]. The data reported here does not support the enzymatic cleavage assumption, but rather gives credence to the shaving hypothesis which proposes that the accelerated loss of membrane bound CD21 occurs in the reticuloendothelial system during the removal of opsonized ICs [32]. Like malaria, the other diseases that are characterized by immune-complex formation such as juvenile arthritis, SLE and Sjögren's syndrome, have reduced levels of sCD21 [35,36]. sCD21 retains the capacity to bind iC3b and CD23 [37], and the reduced levels may thus impair responses that are mediated by these ligands.

C3dg is a downstream product of the complement activation and can be used as a surrogate measure of complement activation. As shown in Figure 1D, children in the SMA group had an increased deposition of C3dg on the surface of B cells compared to the UM group. Increased C3dg deposition is a pointer to increased complement activation during SMA. Because it is the deposition of complement product that mediate interaction of B cell with the macrophages, the increased deposition of complement product in SMAs is responsible for shaving of CD21 [38].

## Conclusions

Results from this study show B-cell involvement in the immunopathology of severe malaria anaemia, mainly involving expansion of mature B-cell population, deposition of C3dg and the loss of both membrane bound and soluble CD21. Interestingly, the loss of membrane-bound CD21 is not through the classical enzymatic cleavage, but probably involves interaction of CD21 loaded with C3dg with cells of reticuloendothelial system, in a manner analogous to removal of C3b bearing CR1 on red blood cells.

## Competing interests

The authors declare that they have no competing interests.

## Authors' contributions

JCK was a graduate student in Jomo Kenyatta University of Agriculture and Technology, Nairobi, Kenya. He conducted all the experiments, helped in data analysis and drafted the manuscript. Both JKM were university supervisors to JCK and assisted in study design and edited the manuscript. JNW designed the study, supervised the work and helped in drafting the final manuscript. All authors have read and approved the final manuscript.
